# Time of HIV diagnosis, CD4 count and viral load at antenatal care start and delivery in South Africa

**DOI:** 10.1371/journal.pone.0229111

**Published:** 2020-02-13

**Authors:** Dorina Onoya, Cornelius Nattey, Nelly Jinga, Constance Mongwenyana, Gayle Sherman

**Affiliations:** 1 Health Economics and Epidemiology Research Office, Department of Internal Medicine, School of Clinical Medicine, Faculty of Health Sciences, University of the Witwatersrand, Johannesburg, South Africa; 2 Department of Paediatrics and Child Health, Faculty of Health Sciences, University of the Witwatersrand and National Institute for Communicable Diseases, a division of the National Health Laboratory Service, Johannesburg, South Africa; CIRCB - Chantal BIYA International Reference Centre for research on HIV/AIDS prevention and management, CAMEROON

## Abstract

**Background:**

Despite the success of prevention of mother to child transmission (PMTCT) program in South Africa, the 30% HIV prevalence among women of childbearing age requires the PMTCT program to be maximally efficient to sustain gains in the prevention of vertical HIV transmission. We aimed to determine the immunologic and virologic status at entry into antenatal care (ANC) and at childbirth among HIV positive women who conceived under the CD4<500 cells/μl antiretroviral therapy (ART) eligibility threshold and universal test and treat (UTT) policies in the Gauteng province of South Africa.

**Method:**

We conducted a retrospective cohort study of 692 HIV positive adult (>18 years) postpartum women who gave birth between September 2016 and December 2017. Demographic, viral load (VL) and CD4 data at ANC start (3–9 months before delivery) and delivery (3 months before/after) were obtained from medical records of consenting women. We compared CD4≥500 cell/μl and viral load (VL) suppression (<400 copes/ml) rates at ANC start and delivery among women with a pre-pregnancy ART, women known HIV positive but with in-pregnancy ART and newly diagnosed women with in-pregnancy ART. Predictors of having a high CD4 and suppressed VL were assessed by log-binomial regression.

**Results:**

Of the 692 participants, 394 (57.0%) had CD4 data and 326 (47.1%) had VL data. Overall women with a pre-pregnancy ART were more likely to start ANC with CD4 count≥500 cell/μl (46.3% vs 24.8%, adjusted risk ratio (aRR) = 1.9; 95% confidence interval (95% CI): 1.4–2.5), compared to newly diagnosed women. This difference was no longer apparent at the time of delivery (aRR 1.2 95% CI: 0.4–3.7). Similarly, viral suppression at delivery was higher among women with pre-pregnancy ART (87.2% vs 69.3%, aRR 1.3, 95% CI: 1.1–1.6) as compared to the newly diagnosed women. Viral suppression rate among newly diagnosed women increased substantially by the time of delivery from 43.5% to 69.3% (p = 0.001).

**Conclusion:**

These results show that pre-pregnancy ART improves immunologic and virologic control during pregnancy and call for renewed efforts in HIV testing, linkage to ART and viral monitoring.

## Introduction

The prevention of mother to child transmission (PMTCT) program is South Africa’s most successful HIV prevention program given the nearly universal coverage and patient uptake of the service [1–4]. Although the ten weeks vertical HIV transmission rate was reported as 0.9% in the 2017/2018 fiscal year [5], as long as one-third of South African women of childbearing age are HIV infected, the PMTCT program must be maximally efficient to sustain gains in the prevention of vertical HIV transmission [1, 6].

In January 2015, the HIV treatment guidelines were updated to increase the cluster of differentiation four (CD4) threshold for ART eligibility for adults to 500 cells/μl and initiate all pregnant and breastfeeding HIV positive women on lifelong antiretroviral therapy (ART) on the day of HIV diagnosis [7]. Furthermore, in 2016, South Africa adopted the World Health Organization (WHO)’s HIV universal test and treat (UTT) policy, effectively expanding ART access to non-pregnant HIV positive women with CD4 count above 500 cells/μl [8]. Viral load (VL) and CD4 count measures are critical markers of immunologic wellbeing and viral control in persons living with HIV [9]. Higher viral load and lower CD4 cell count increase the vulnerability to opportunistic infections as well as the risk of vertical and sexual HIV transmission [10]. Moreover, VL suppression impacts positively on the health of both mother and foetus by controlling HIV disease progression [11, 12]. Therefore, sustained pre-pregnancy ART adherence and viral suppression among HIV positive women will remove some of the pressure on the antenatal care (ANC) components of the PMTCT program and improve the efficiency of healthcare providers in preparing women who are diagnosed during pregnancy for in-pregnancy and postpartum ART adherence and retention in care.

Among HIV positive women who know their HIV status and initiate ART before a pregnancy, pre-pregnancy ART experience has the potential to increase the demand for ANC and PMTCT services though appropriate education on the risk of vertical HIV transmission during pregnancy. While fast-tracking HIV positive women with pre-pregnancy ART through the first ANC visit and focusing on adherence counselling may be warranted [7, 13], as the population of women with pre-pregnancy ART experience changes, it is essential to understand the associated changes in CD4 count and VL suppression both at entry into ANC and at delivery, as well as their implication on long-term viral control and retention outcomes [14]. However, studies conducted before 2013, when pre-pregnancy ART in South Africa was reserved for patients with CD4 count under 350 cell/μl, showed that women with pre-pregnancy ART were at higher risk of virologic failure during pregnancy and postpartum compared to women with in-pregnancy ART [15–17]. However, women who conceived on ART were more likely to be retained in HIV care compared to women with in-pregnancy ART [15]. We have recently shown that South African women who initiated pre-pregnancy ART under the CD4<500 ART eligibility threshold (January 2015 to September 2016) do not start ANC care earlier than their HIV naive counterparts [18]. Furthermore, there is limited evidence to suggest that, under the CD4<500 eligibility criteria and now UTT, HIV positive women with pre-pregnancy ART enter ANC and complete the pregnancy period with higher immunological status or controlled viremia compared to those who initiate ART during pregnancy.

In this study, we aimed to determine the immunologic and virologic status at entry into ANC and at childbirth among HIV positive women who conceived under the CD4<500 cells/μl ART eligibility threshold and UTT policies. We further aimed to compare the proportion of women with CD4≥500 cells/μl and virologic suppression (VL<400 copies/ml) by the timing of HIV diagnosis and ART initiation.

## Materials and methods

### Study design and population

We conducted a retrospective cohort study of 692 HIV positive adult (≥18 years) postpartum women who gave birth between September 2016 and December 2017. Participants were recruited after their postnatal visit (scheduled three to six days after delivery) at Midwife Obstetric Units (MOUs) in peri-urban townships of the Tshwane, Ekurhuleni and Johannesburg Metropolitan districts in the Gauteng Province of South Africa. MOU facilities provide primary-level obstetric services including the delivery and immediate post-natal components of the PMTCT care cascade [7]. Pregnant women who present at MOUs with complications are referred to secondary-level hospitals for delivery and further medical treatment when necessary. However, all women with uncomplicated deliveries (not needing post-natal hospitalisation) are referred back to the MOU for the postnatal assessment before being further referred to primary health care (PHC) clinics for postpartum care.

HIV positive women were recruited consecutively from October 2016 to January 2018, via referrals from nurses at participating MOU facilities and interviewed on the day of study enrolment. Study staff screened all referred patients for eligibility and obtained written informed consent using an information sheet administered in the participant’s preferred language (available in English, Sotho and Zulu). Women were excluded from the study if they were under 18 years of age, had delivered more than four weeks prior to study enrolment, were psychologically unable or too sick to participate. Eligible and consenting women completed an interviewer-administered questionnaire (S1 File) on the day of study enrolment.

The sample was determined based on the fact that 30% of South Africa pregnant women who test HIV positive during antenatal care had a previous HIV positive diagnosis and only 40% of South African women attend ANC before the recommended 14 weeks gestation [7, 19, 20]. We sought as total sample of 360 HIV positive women to detect a 20% difference in the timing of ANC initiation indicators between women with or without a prior HIV positive diagnosis during pregnancy, using an alpha (α) of 0.05, 80% power and a 1:1 allocation ratio. In order to detect changes in health status (VL and CD4) between antenatal care and delivery, we doubled the sample size in expectation of missing laboratory indicators. Further details about the study procedures were previously reported [21, 22].

The South African guideline for HIV treatment and monitoring for pregnant and postpartum women recommends CD4 monitoring at ANC start/ HIV diagnosis and then 12 months thereafter to monitor patients’ immune response to ART [7]. HIV viral load (VL) monitoring in pregnancy occurs from pregnancy diagnosis/ANC start (HIV positive women who are already receiving ART) or three months after ART initiation for women with in-pregnancy HIV diagnosis, and then at three-monthly intervals (3, 6, 12, 18 and 24 months) throughout pregnancy and breastfeeding [7]. Generally, whole blood samples are collected from HIV diagnosed patients at the site of care and sent to a central laboratory of the National Health Laboratory Services (NHLS) for testing. Viral load determination at the NHLS are done using the Abbott m2000 RealTime HIV-1 Viral Load System [23]. CD4 counts are determined by immune-phenotyping using the PanLeucogate (PLG) CD4 flow cytometry assay [24]. We did not manage the blood collection or the specimen testing but obtained retrospective data on VL and CD4 count at ANC initiation (three to nine months before delivery) and delivery (90 days before or after child-birth) from the NHLS records of consenting women.

### Outcome variable

The primary outcome variable for this analysis was HIV viral suppression, defined as VL<400 copies/ml. The secondary outcome variable for this analysis was high CD4 count defined as CD4>500 cells/μl. These were determined at ANC start (first value recorded 3–9 months before delivery) and delivery (value closest to the date of delivery, recorded three months before/after delivery)

### Exposure variables

We compared the proportion of participants with high CD4 count and viral suppression at ANC start and delivery by the timing of ART categorised as:

1. Women with a pre-pregnancy ART
2. Women with known HIV positive status at ANC start but in-pregnancy ART
3. Women who were diagnosed with HIV and initiated ART in-pregnancy

Additional variables assessed included socio-demographic factors, measured at delivery, including age, highest level of education completed, marital status, employment status and work times (all-day, shift-work) and income-source. We also assessed factors related to ANC attendance, location and type of housing, whether the latest pregnancy was planned, number of child dependents (own and others'), support during pregnancy, and expected childcare support postpartum.

### Statistical analysis

**Fig 1:**
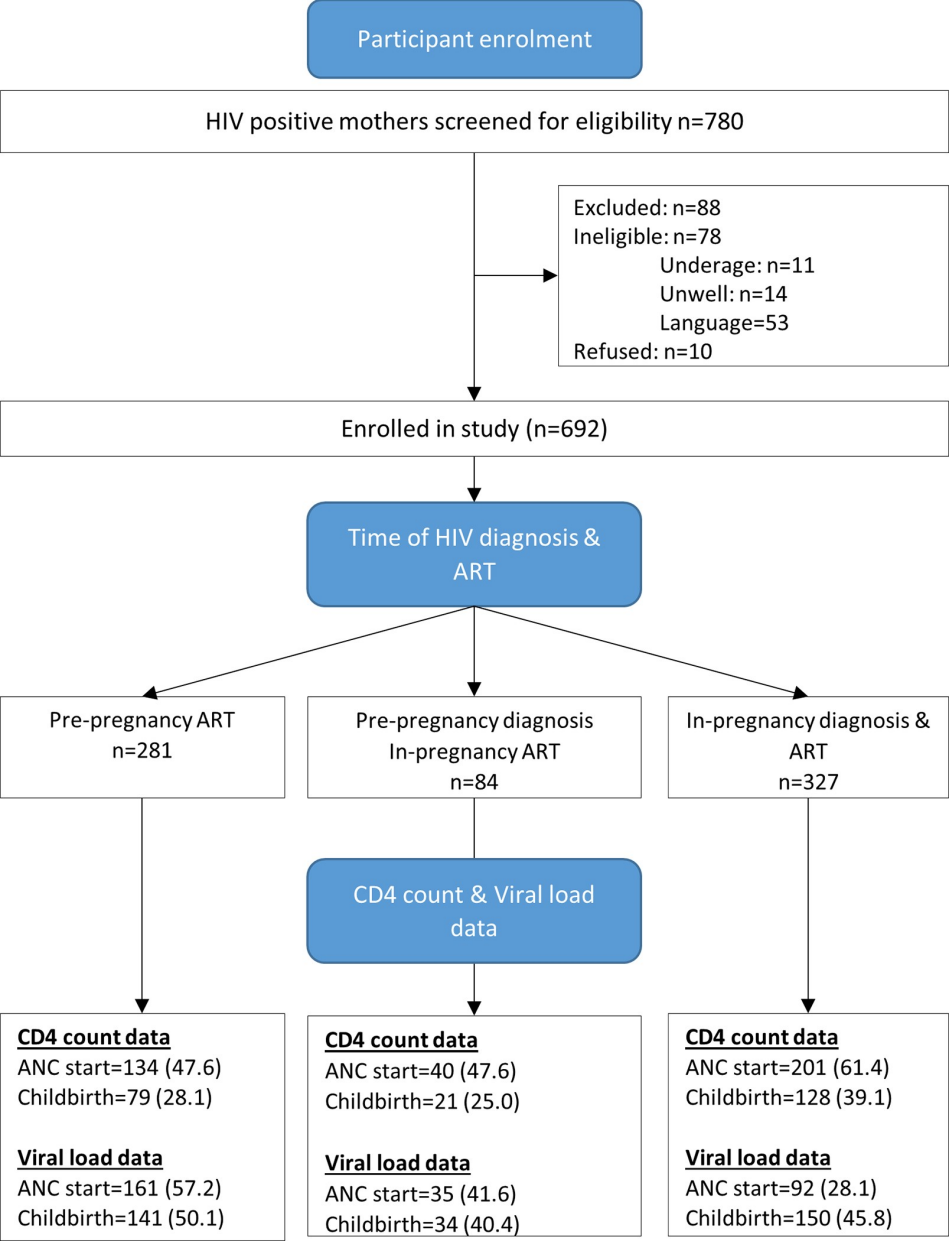
CONSORT diagram of retrospective cohort and data completeness.

Continuous variables were described using means, median, standard deviations and interquartile range (IQR). A t-test was used to test for significance. Categorical variables were summarized using percentages and were assessed by log-binomial regression models. Adjusted Log-binomial regression model was used to estimate risk ratio (RR) for predictors of having a CD4≥500 or suppressed VL at ANC initiation and delivery and the associated 95% confidence intervals (95% CIs) after adjusting for potential confounders. Considering that the natural VL progression in untreated HIV positive patients and the fact that women with in-pregnancy HIV diagnoses do not get VL tests at ANC start (first test at three months post-ART), we only assess predictors of viral suppression at ANC start among women with a pre-pregnancy ART. Participants who were missing VL data were excluded from the VL specific analysis. Similarly, participants who were missing CD4 data were excluded from CD4 specific analyses. Sample sizes for each analysis are shown in Fig 1. Variables with a univariate p-value <0.1 and priory variables of importance were included in the multivariate model. Significance level was set at p = 0.05 for the multivariate log-binomial regression model. Data analysis was conducted using STATA version 14 (StataCorp, College Station, TX). Ethics approval for the study was obtained from the Wits Human Research Committee (HREC No. M151041).

## Results

### Socio-demographic characteristics

Overall, 88.7% of referred women participated in the study, 53/88 were excluded due to language problems while the remaining 35/88 could not participate because of illness or age factors (Fig 1).

Table 1 summarises characteristics of 692 women at the timing of HIV diagnosis. About 365 (53%) were known HIV positive at ANC start, and 327 (47%) were diagnosed during the latest pregnancy. Nearly half (45.3%) of women with pre-pregnancy HIV diagnosis had planned the most recent pregnancy. The proportion of planned pregnancies was similar among newly diagnosed women (44.0%). Overall, 56% (394/692) had CD4 data at ANC start and 33% (229/692) at childbirth. Among women with pre-pregnancy diagnosis, 281 (76%) had pre-pregnancy ART experience. Among women in the pre-pregnancy ART group, 57.3% had VL data at ANC start. In total, 47% (326/692) participants had VL data at childbirth (Fig 1).

**Table 1:** Characteristics of HIV positive women attending postpartum care in Gauteng South Africa.

	Pre-pregnancy ART	Pre-pregnancy HIV diagnosis In-pregnancy ART	In-pregnancy HIV diagnosis & ART	Total
	No.(%)	No.(%)	No.(%)	No.(%)
	N = 281	N = 84	N = 327	N = 692
**Age (years)**				
18–25	34 (12.1)	21 (25.0)	97 (29.8)	152 (22.0)
26–30	64 (22.7)	27 (32.1)	103 (31.6)	194 (28.1)
31–35	98 (34.9)	23 (27.4)	81 (24.8)	202 (29.23)
>35	85 (30.3)	13 (15.5)	45 (13.8)	143 (20.7)
**Highest level of education**				
Tertiary level	32 (11.4)	5 (6.0)	50 (15.3)	87 (12.6)
Matric	64 (22.9)	9 (10.7)	86 (26.4)	159 (23.0)
High school	169 (60.4)	62 (73.8)	180 (55.2)	411 (59.6)
Primary school or less	15 (5.4)	8 (9.5)	10 (3.1)	33 (4.8)
**Employment**				
Employed	115 (40.9)	21 (25.0)	131 (40.2)	267 (38.6)
Unemployed (not job hunting)	30 (10.7)	20 (23.8)	48 (14.7)	98 (14.2)
Unemployed (job hunting)	136 (48.4)	43 (51.2)	147 (45.1)	326 (47.2)
**Marital status**				
Married	59 (21.1)	14 (16.7)	51 (15.7)	124 (18.0)
In a relationship	207 (73.9)	69 (82.1)	258 (79.4)	534 (77.5)
Not in a relationship	14 (5.0)	1 (1.2)	16 (4.9)	31 (4.5)
**Participant lives with**				
With partner/spouse	183 (65.8)	66 (80.5)	202 (62.7)	451 (66.1)
Parents/relatives	68 (24,5)	11 (13.4)	101 (31.3)	180 (26.4)
Alone/with children	27 (9.7)	5 (6.1)	19 (5.9)	51 (7.5)
**Monthly income (respondent)**				
< R2000	31 (27.0)	7 (33.3)	22 (16.8)	60 (22.5)
R2000-R5999	74 (64.4)	13 (61.9)	92 (70.2)	179 (67.0)
> = R6000	10 (8.7)	1 (4.7)	17 (13.0)	28 (10.5)
**Was the latest pregnancy planned?**				
Yes	127 (45.2)	42 (50.0)	144 (44.2)	313 (45.3)
No	154 (54.8)	42 (50.0)	182 (55.8)	378 (54.7)
**How many children do you have (older than this baby)?**				
0 children	19 (6.8)	12 (14.8)	60 (18.8)	91 (13.4)
1–2 children	209 (74.9)	57 (70.4)	223 (69.9)	489 (72.0)
>2 children	51 (12.3)	12 (14.8)	36 (11.3)	99 (14.6)
**CD4 count at antenatal care**				
<500	79 (28.1)	28 (33.3)	151 (46.2)	258 (37.3)
≥500	68 (24.2)	16 (19.1)	50 (15.3)	134 (43.4)
Missing	134 (47.7)	40 (47.6)	126 (38.5)	300 (43.3)
**CD4 count at delivery**				
<500	41 (14.6)	12 (14.3)	85 (26.0)	138 (19.9)
≥500	38 (13.5)	9 (10.7)	43 (13.2)	90 (13.0)
Missing	202 (71.9)	63 (75.0)	199 (60.8)	464 (67.1)
**Viral load at antenatal care**				
≥400	37 (13.1)	15 (17.9)	52 (15.9)	104 (15.0)
<400	124 (44.1)	20 (23.8)	40 (12.2)	184 (26.6)
Missing	120 (42.7)	49 (58.3)	235 (71.9)	404 (58.4)
**Viral load at delivery**				
≥400	18 (6.4)	7 (8.4)	46 (14.1)	91 (13.2)
<400	123 (43.8)	27 (32.1)	104 (31.8)	234 (33.8)
Missing	140 (49.8)	50 (59.5)	177 (54.1)	367 (53.0)
**Aneamia**				
Normal	85 (60.3)	24 (60.0)	69 (43.7)	178 (52.5)
Aneamic	56 (39.7)	16 (40.0)	89 (56.3)	161 (47.5)

The median age was 30 years (IQR: 26–35) at childbirth, 124 (18%) were not married (77.5% of the women were in non-marital relationships), and nearly two thirds (66.1%) lived with a partner or spouse. Newly diagnosed women were a little younger (median 29; IQR: 25–33 vs 33; IQR:18–36; Ranksum p<0.01) than known HIV positive women. Over 60% of women had completed high school education with the majority (61.4%) being unemployed. About 72% had at least one child older than the most recent baby. There were no significant differences in the socio-demographic characteristics of women with in-pregnancy HIV diagnosis compared to those with pre-pregnancy HIV diagnosis.

### Comparing high CD4 rates at ANC and delivery

**Fig 2:**
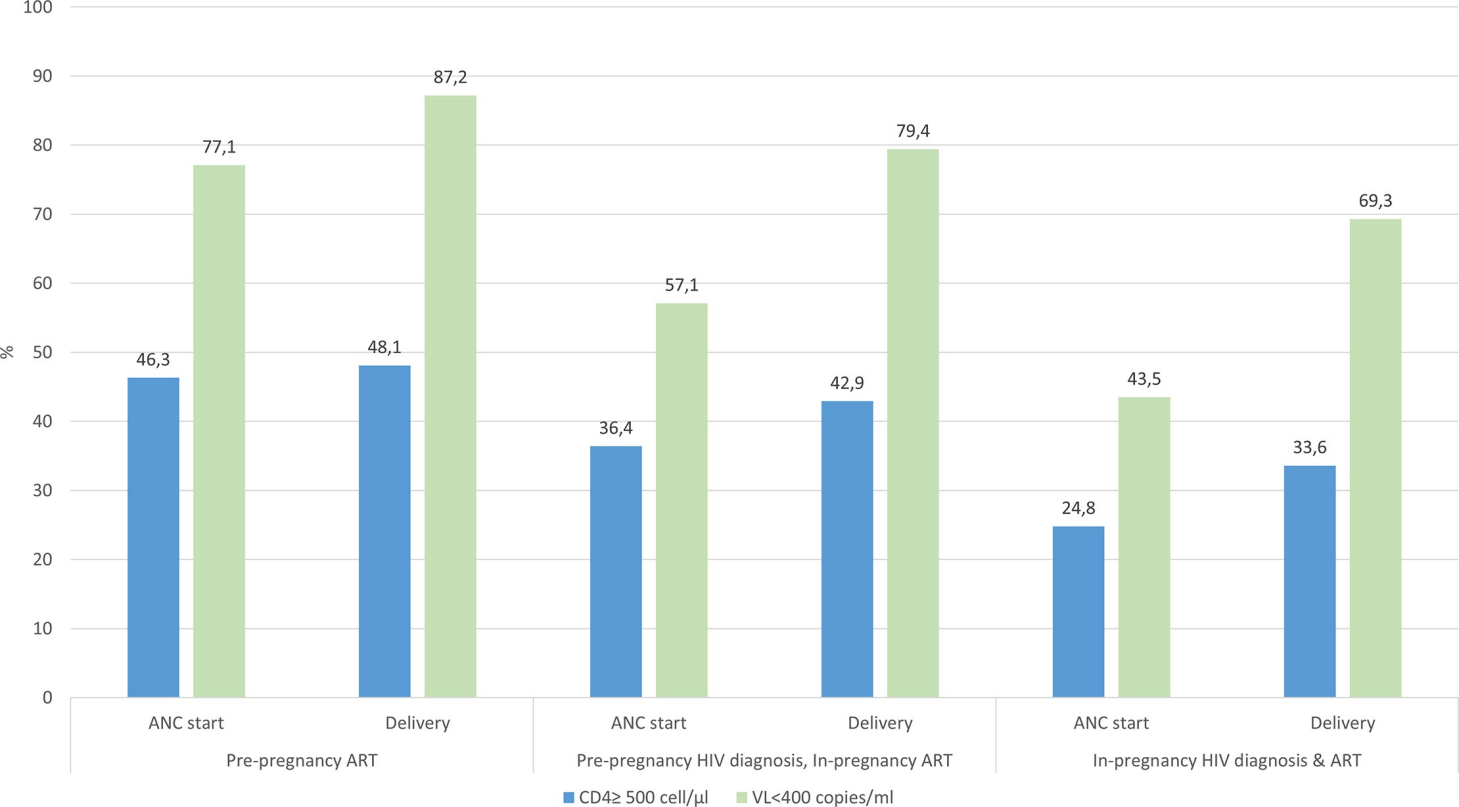
High CD4 count and viral suppression at ANC start and childbirth among HIV positive women by the timing of HIV diagnosis and ART initiation.

We compared high CD4 count (≥500 cells/μl) rates at ANC initiation and delivery by the timing of HIV diagnosis and ART initiation (Fig 2, Table 2).

**Table 2:** Log-binomial regression models for high CD4 Count at ANC start and delivery among HIV positive women attending postpartum care in Gauteng South Africa.

	ANC start	Delivery
Variables	Adjusted RR (95% CI)	Adjusted RR (95% CI)
**Timing of HIV diagnosis**		
In-pregnancy HIV diagnosis & ART	1	1
Pre-pregnancy ART	1.9 (1.3–2.5)	1.4 (0.7–3.0)
Pre-Pregnancy HIV diagnosis, in-pregnancy ART	1.3 (0.7–2.3)	1.5 (0.5–4.1)
**Age**		
18–25	1	1
26–30	1.2 (0.8–1.8)	1.2 (0.8–1.8)
31–35	1.5 (0.9–2.2)	0.9 (0.6–1.5)
>35	1.4 (0.9–2.1)	0.8 (0.4–1.7)
**Education**		
Tertiary level	1	1
Matric	1.4 (0.9–2.3)	1.5 (0.8–2.6)
High school	1.0 (0.6–1.6)	1.3 (0.8–2.3)
Primary school or less	1.2 (0.4–2.6)	0.9 (0.3–2.8)
**Marital status**		
Married	1	1
In a relationship	0.9 (0.6–1.2)	1.1 (0.6–1.9)
Not in a relationship	0.8 (0.5–1.1)	1.2 (0.7–2.1)
**Participant lives with**		
With partner/spouse	1	1
Parents/relatives	0.7 (0.5–1.0)	1.2 (0.8–1.7)
Alone/with children	1.6 (1.1–2.3)	0.9 (0.4–1.8)
**Employment**		
Employed	1	1
Unemployed (not job hunting)	1.3 (0.8–1.9)	1.1 (0.5–2.1)
Unemployed (job hunting)	0.9 (0.7–1.3)	1.5 (1.1–2.2)
**What is your monthly income?**		
< R2000	1	1
> = R2000-R5999	1.1 (0.6–1.9)	0.9 (0.5–1.7)
> = 6000	1.1 (0.4–2.8)	0.6 (0.1–2.1)
**Was this pregnancy Planned?**		
No	1	
Yes	1.0 (0.8–1.3)	NI
**How many children do you have (older than this baby)?**		
0 children	1	1
1–2 children	0.9 (0.6–1.4)	0.4 (0.2–0.8)
>2 children	1.1 (0.7–1.8)	1.3 (0.4–2.5)
**Anaemia status**		
Normal	1	
Anaemia	0.9 (0.6–1.2)	NI

NI: not included in multivariate model; RR: Risk ratio; aRR: Adjusted risk ratio

Overall, women with a pre-pregnancy ART (46.3%) were more likely to present at ANC with CD4≥500 cell/μl compared to newly diagnosed women (24.8%) (aRR 1.9, 95% CI: 1.3–2.5). At ANC initiation, there was no difference in the immunologic status of newly diagnosed women and women who were known HIV positive but only started ART in-pregnancy (36.4%) (aRR 1.3, 95%CI: 0.7–2.3). These differences were no longer apparent at the time of delivery (48.1%, 42.8% and 33.5% respectively). There was, however, an increase in high CD4 count rates among women who were diagnosed but not on ART before ANC (36.4% to 42.8%) and those who were diagnosed in-pregnancy (24.9% to 33.6%).

Neither younger age, level of education, marital status, employment status, parity, family planning, nor mother's income explained the association between the timing of HIV diagnosis and having a CD4>500 at ANC start (Table 2). However, women who lived alone or only with children were more likely to start ANC with a higher CD4 count (RR 1.6, 95%CI: 1.1–2.3) compared to women who lived with a partner/spouse. Although newly diagnosed women were nearly twice as likely to be anaemic at ANC start, women’s period-specific anaemia status was not associated with high CD4 count at ANC start (aRR 0.9; 95% CI: 0.6–1.2). Unemployed participants who were job hunting were more likely to have a high CD4 count at delivery (RR = 1.5; 95% CI: 1.1–2.2) compared to employed women. Also, primiparous women were more likely to have a high CD4 count at delivery compared to women wo had at least 1 additional child (aRR 2.5, 95% CI: 1.3–5.0).

### Comparing VL suppression rates at ANC and delivery

**Fig 3:**
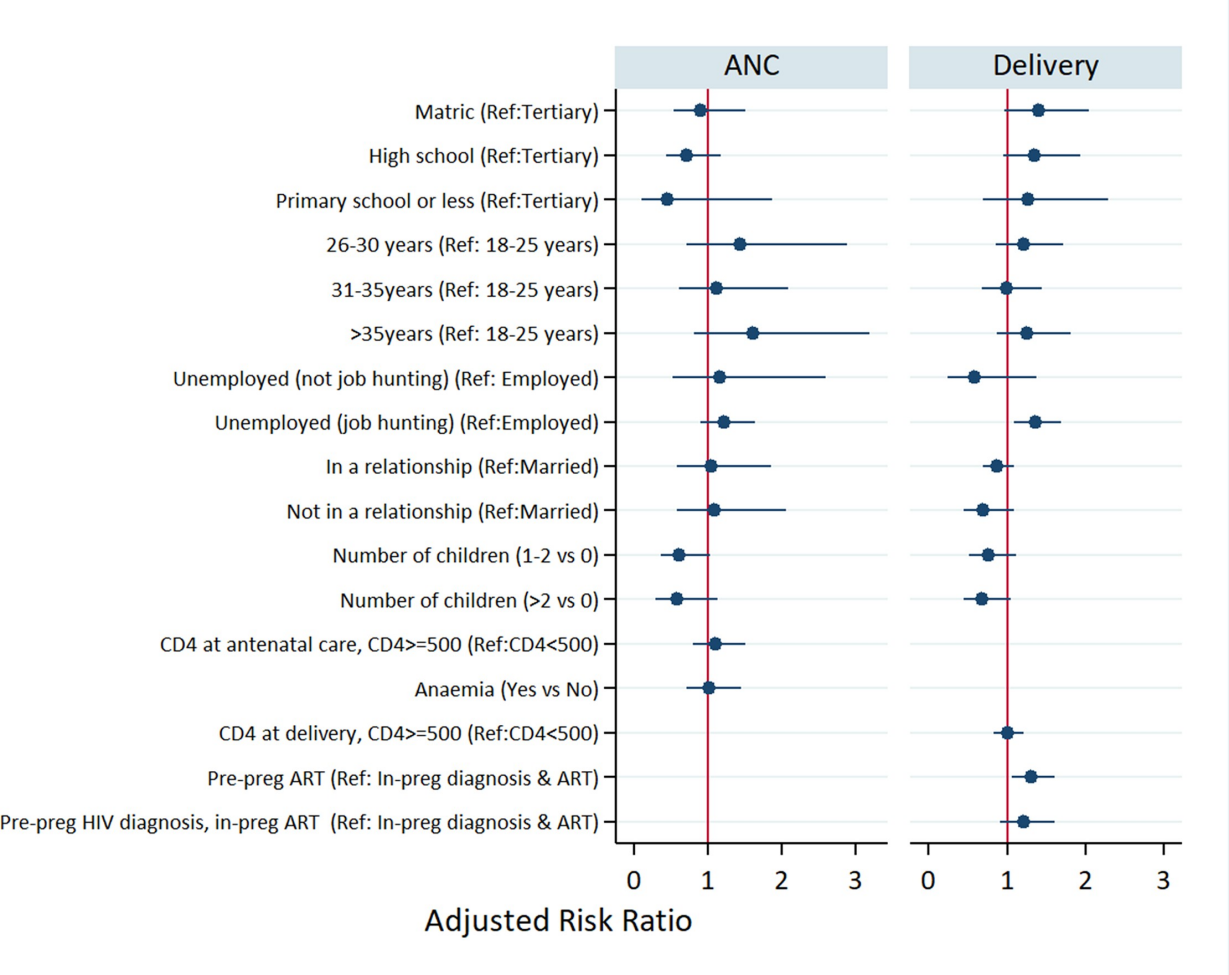
Multivariate log-binomial regression models for viral suppression at ANC star (only women with pre-pregnancy ART) and at childbirth (all) among HIV positive.

We also compared VL suppression (<400 copies/ml) rates around ANC initiation and delivery across the three groups of women (Fig 2). Similarly, to CD4 count patterns, a higher proportion of women with pre-pregnancy ART had a suppressed VL at ANC start compared to women with pre-pregnancy diagnosis but in-pregnancy ART, and the newly diagnosed group (77.1%, 57.1% and 43.5% respectively). In multivariate log-binomial models (Fig 3), although viral suppression rates among newly diagnosed women (43.5% to 69.3%) increased substantially by the time of delivery, those with a pre-pregnancy ART (87.2%) remained 30% more likely to be virally suppressed at delivery (aRR 1.3, 95% CI: 1.1–1.6).

Among those with pre-pregnancy ART, being multiparous (aRR 0.3 for 1–2 children vs no child; 95% CI: 0.1–0.9) and being in a non-marital relationship (aRR 0.3 for being unmarried vs married, 95% CI: 0.1–1.0) decreased the likelihood of viral suppression at ANC start by 70%. Additionally, among women with pre-pregnancy ART, being unemployed and job hunting was associated with a higher likelihood of viral suppression at ANC start (aRR 1.3; 95% CI: 1.1–1.7) compared to being employed. Having a high CD4 count was not associated with viral suppression at both time points.

## Discussion

We aimed to assess immunologic status (CD4 >500) at the start of ANC service and at childbirth, comparing HIV positive women with pre-pregnancy ART to women known HIV positive but with in-pregnancy ART and women who were diagnosed and started ART in-pregnancy. We then examined predictors of starting ANC with suppressed VL among women with pre-pregnancy ART. Finally, we assessed viral suppression at childbirth by the timing of HIV diagnosis and ART initiation.

We found that nearly half of the HIV positive pregnant women had planned their latest pregnancy and knew their HIV status before ANC. Also, over 70% of women who knew their HIV positive conceived on ART. This high fertility intention among women who started ART before a pregnancy was also demonstrated among HIV positive women in the Western Cape Province of South Africa [25]. Furthermore, women with pre-pregnancy ART entered ANC with higher immunological status compared to women who were undiagnosed or diagnosed but not on ART before the most recent pregnancy, but this difference faded at the end of the pregnancy. Moreover, we found that women with pre-pregnancy ART had higher viral suppression rates at childbirth compared to women with in-pregnancy ART.

These results are in contrast with data from the International Maternal, Paediatric, Adolescent AIDS Clinical Trials (IMPAACT) Group, 2002–2011 group analysis in which more women with pre-pregnancy HIV diagnosis had detectable VL at delivery as compared to women diagnosed with HIV during pregnancy [26, 27]. Similarly, studies in South Africa, under the CD4<350 ART threshold [28], showed that women with pre-pregnancy ART were at higher risk of virologic failure during pregnancy and postpartum compared to women with in-pregnancy ART [15–17]. In this study, low CD4 at ART start was also an important predictor of virologic failure both in-pregnancy and postpartum.

The estimated 72% overall VL suppression at childbirth in our sample is lower than the 88% reported national ART suppression rates [29]. Though the 87% suppression at delivery among women with pre-pregnancy ART is encouraging, at least 20% of women who were known positive but not on ART at ANC start had persistent viremia throughout the pregnancy. A recent study showed that HIV viremia at ANC start among known HIV positive pregnant women accounts for 25% of vertical transmission in South Africa [30]. Among women who started pre-pregnancy ART, promoters of VL suppression at entry into ANC were seeking employment, being a first-time mother and being married. The association between viral suppression at entry into ANC with unemployment and being married highlight the need for free time and partner/social support in seeking HIV care and ART adherence [31, 32].

Similar to previous studies, we found that multiparity decreased the likelihood of viral suppression at childbirth, possibly because the increased childcare demands challenge ART adherence [33, 34]. Multiparity is also an important determinant of later ANC and ART initiation during pregnancy, which may explain its association with HIV viremia at delivery [22].

Younger age at pregnancy, being married and attending at least two postpartum clinic visits have been cited as important for postpartum retention and viral suppression among HIV positive women in South Africa [35].

Currently, the risks of post-partum virologic failure and retention in care in the UTT era are unclear. Nevertheless, these results highlight the benefit of expanding pre-pregnancy ART access to women in higher CD4 count groups in ensuring safer pregnancies [32]. These results not only highlight the importance of intensifying pre-pregnancy diagnosis and ART initiation but also the need to expand adherence support to improve and sustain viral suppression among women who start ART in-pregnancy.

Our findings are limited by the context in which the study was conducted and may not be generalizable to the rest of the country. Additionally, women reported personal and contextual data at the time of childbirth, which may differ from their circumstances at the time of conception and during the pregnancy. Although, there is evidence that study participants attended health facilities for delivery and the vast majority had attended ANC services, after an extensive national search on the NHLS database, over half of the women lacked CD4 count and VL data during pregnancy. While, the lack of a unique national patient identifier in our healthcare service, probably contributed to difficulties in locating laboratory, particularly VL results, a 2018 study concluded that VL results were missing from patient records mainly because the tests were not requested when they were due [36]. Therefore, the results are limited to patients for whom CD4 and VL assessment were ordered [36]. However, our data highlights the value of pre-pregnancy ART in reducing the risk of MTCT directly via VL suppression and indirectly via higher CD4 count i.e. mother’s better health status at ANC start and delivery. Further studies with sufficient data are needed to better depict changes in viral load at ANC start and delivery

In conclusion, the study results show that HIV positive women with pre-pregnancy ART are better able to sustain high immunologic status and virologic control in pregnancy compared to women who are ART naïve at ANC start. These results are encouraging and support the efforts to expand pre-pregnancy ART and viral monitoring in South Africa.

## Supporting information

S1 File Study questionnaire. (DOC)
