# Supplementary material for: Time of HIV diagnosis, CD4 count and viral load at antenatal care start and delivery in South Africa
Source: PLoS One. 2020 Feb 13;15(2):e0229111. doi: 10.1371/journal.pone.0229111 (PMC7018033; doi:10.1371/journal.pone.0229111)
Supplement: S1 File — (DOC) [file pone.0229111.s001.doc]

**Part B.** **Baseline Questionnaire (HIV pos. participant)**

| **DEMOGRAPHICS**  *Interviewer reads: Now I would like to ask you some questions concerning the situation you are currently in. Please try to answer the questions as accurately as possible. This first series of questions are about you, your family and your work.* | | |
| --- | --- | --- |
| DM1 | Race | African 1  White 2  Coloured 3  Indian/Asian 4 |
| DM2 | Please indicate your primary language |  |
| DM3 | What is the highest standard or grade that you have completed? | Never attended school 1  Primary school 2  High school 3  Completed matric 4  Short course/ certificate 5  University/college 6  Other 7 |
| DM4 | What is your current marital status? | Single, no current partner 1  In a relationship (not living together) 2  In a relationship (living together) 3  Married 4  Divorced/ Separated 5  Widowed 6 |
| DM5 | Do you live in | Own home 1  Family's home 2  Relative's home 3  Friend's home 4  Other 5 |
| DM6 | Who do you live with? | Alone 1  With a partner/ spouse 2  Children 3  Parent/ relatives 4  Friends 5  Other 6 |
| DM7 | How many other adults live in your household (excluding yourself)? | Adults (18 years or older): |
| DM8 | How many children live in your household | Children (younger than 18 years): |
| DM9 | What type of house do you live in? | House or brick structure in  a separate stand or Yard 1  Flat in a block of flats 2  House/room/flat in  someone else's house or yard 3  Informal dwelling or shack 4 |
| DM10 | In what area do you currently live? |  |
| DM11 | How many years have you lived in your current house? | < 1 year 1  1-5 years 2  >5 years 3  Don’t know 4 |
| DM12 | Is your current house your primary house | No 0  Yes**🡪** **DM14** 1 |
| DM13 | Where is your primary house? | Rural area 1  Another province 2  Another country 3 |
| DM14 | What is your current employment status? | Employed (full/part-time) 1  Self-employed 2  Unemployed (job hunting) **🡪** **DM17** 3  Unemployed (not job hunting) **🡪** **DM17** 4  Sick/ disabled**🡪** **DM17** 5  Pensioner**🡪** **DM17** 6  Student**🡪** **DM17** 7 |
| DM15 | When do you usually work? | Mornings only 1  All day 2  Afternoons only 3  Night only 4  Shifts change 5 |
| DM16 | What is your current MONTHLY income? | Less than R1999 1  R2000-R5999 2  R6000-R9999 3  R10000-R15999 4  16000-R20000 5  More than R20001 6 |
| DM17 | Where do you get MOST of your money from? | Paid job, salary or business 1  Government grant 2  Spouse/ partner 3  Parents/ relatives 4  Friends 5  Other 6 |
| DM18 | In the past 12 months, were there times when members of your household went hungry because there was not enough food in the house? | YES 1  NO 0 |

| **DEPENDANTS (older)**  *Interviewer reads: Now I would like to ask you some questions concerning children that you care for other than this new baby.* | | | | |
| --- | --- | --- | --- | --- |
| DP1 | How many children do you have (older than this new baby/ies)? | (if 0, **🡪** **DP3**) | | |
| DP2 | How old is the oldest child? | ______years | ______months |  |
|  | How old is the youngest child? | ______years | ______months |  |
| DP3 | How many of these children are HIV positive | if 0, **🡪DP5**) | | |
| DP4 | Are any of the children taking HIV treatment medications? | Yes 1  No 0  Don’t know 98 | | |
| DP5 | How many children other than your own do you care for and live with? |  | | |
| **DEPENDANTS (New baby/ies)**  *Interviewer reads: Now I would like to ask you some questions concerning this new baby (babies if twins)* | | | | |
| DP6 | Do you know the HIV status of your baby | Yes - Positive 1  Yes - Negative**🡪DP10** 2  No 3 | | |
| DP7 | Is your baby receiving ARV | Yes**🡪DP14** 1  No 2  Don’t know 3 | | |
| DP8 | If Not, why is the baby not on ARV? | Refused to give consent 1  It was not offered**🡪DP14** 2  Other (specify) **🡪DP14** 3 | | |
| DP9 | Why did you refuse to give consent for ARV**🡪DP14** | I am not taking ARV 1  Partner refused 2  Traditional/religious reasons 3  Other (specify) 4 | | |
| DP10 | Why do you not know your child’s HIV status | Refused to provide consent for test 1  I did not know about the test**🡪DP12** 2  I did not get the test results**🡪DP12** 3  Other (Specify) **🡪DP12** 4 | | |
| DP11 | What was the reason for refusing to consent for a HIV test for the baby | Afraid baby will be positive 1  Partner refused 2  Traditional/religious reasons 3  Other 4 | | |
| DP12 | Do you intend to test the baby for HIV in the future | No**🡪DP14** 1  Yes 2 | | |
| DP13 | When do you intend to test the baby/get the results | Next week 1  Next visit 2  When I am ready 3  Not sure when 4 | | |
| DP14 | What is your feeding plan for the baby | Exclusive breastfeeding 1  Mixed feeding (breast and formula) 2  Only formula 3 | | |
| **SUPPORT DURING PREGNANCY AND CHILD CARE** | | | | |
| SP1 | Was this last pregnancy planned? | Yes 1  No 0 | | |
| SP2 | How involved was the baby’s father in the pregnancy? | Very involved 1  Somewhat involved 2  Not at all involved 3 | | |
| SP3 | Who would you consider to be your greatest support base during this pregnancy? | Partner 1  Baby’s father (if not partner) 2  Family members 3  Friends 4  Other 5 | | |
| SP4 | Who in your opinion will help the most in the care of your baby? | Partner 1  Baby’s father (if not partner) 2  Family members 3  Friends 4  Other 5 | | |

| **ACCESS TO CLINICS** | | |
| --- | --- | --- |
| AC1 | Did you see anyone for antenatal care for this pregnancy? | No**🡪** **AC14** 0  Yes 1 |
| AC2 | Who did you see? | Doctor 1  Nurse/Midwife 2  Traditional birth attendant 3  Other 4 |
| AC3 | Where did the first antenatal visit take place? | Public hospital/Clinic 1  Mobile Clinic 2  Private hospital/Clinic 3  Other private medical 4  Other 5 |
| AC4 | How many weeks pregnant were you when you first received antenatal care for this pregnancy? | Weeks |
| AC5 | What is the name of the clinic where you receive health care during your last pregnancy? | . |
| AC6 | Did you attend antenatal care at your usual clinic (where you received health care before becoming pregnant)? | No 0  Yes**🡪** **AC9** 1 |
| AC7 | If you did not receive antenatal care from your usual clinic, why change clinics? | Referred 1  Better care 2  Closer to work 3  Other 4 |
| AC8 | If you received antenatal care at a different clinic, what is the name of this clinic? |  |
| AC9 | How did you get to the clinic for antenatal care? *Circle all that apply*. | On foot 1  By taxi/ bus 2  By car (I drove/ got a lift) 3  Other 4 |
| AC10 | How long did it take you to travel from home/ work to the clinic (**ONE WAY**)? |  |
| AC11 | Did you have to pay anything to travel to the clinic today? | No**🡪 AC13** 0  Yes 1 |
| AC12 | How much did you pay for **ONE WAY**? |  |
| AC13 | Did you take leave from work to attend your antenatal care visits? | Yes (paid leave available) 1  Yes (unpaid) 2  No (no need-employed) 3  No (unemployed) 4  No (self-employed) 5 |
| AC14 | What was the most important reason for not attending antenatal care before the birth of your baby? | Did not know that I should 1  Clinic was too far 2  No money for transport 3  Could not take time off work 4  I did not think it was necessary 5  Other (please specify): 6  Other: |
| **POST-PARTUM MOBILITY** | | |
| PM1 | Now that you have delivered, will you spend the next 24 months in your current home? | Yes**🡪EH1** 1  No 2  Don’t know 3 |
| PM2 | Why will you move after delivery? | Tradition 1  Better support 2  Other (specify) 3 |
| PM3 | If no, where will you go? | To my family home 1  To stay with a friend 2  To stay with a relative 3  Other (please specify): 4 |
| PM4 | In what town & Province will you go to? | Town:  Province: |
| PM5 | What clinic will you go to for follow-up visits? |  |
| PM6 | Who will take the baby for follow up visits | Myself 1  Relative (specify) 2  Other (specify) 3 |
| **EXPERIENCE OF HIV CARE AND TREATMENT** | | |
| EH1 | When did you find out that you were HIV positive? | | D | D | M | M | Y | Y | Y | Y | | --- | --- | --- | --- | --- | --- | --- | --- | |
| EH2 | Were you pregnant when you *first* found out that you were HIV positive? | No 0  Yes 1 |
| EH3 | Were you already receiving HIV treatment (taking ARV) before your latest pregnancy? | No 0  Yes 1 |
| EH4 | Did you go to the clinic to check you CD4 count in the year before becoming pregnant? | No 0  Yes 1 |
| EH5 | When did you start taking ARV pills? | | D | D | M | M | Y | Y | Y | Y | | --- | --- | --- | --- | --- | --- | --- | --- | |
| EH6 | Did your doctor change your ARV when you became pregnant? | No 0  Yes 1  Started ART during pregnancy 2 |
| EH7 | Can you describe the ARVs that you are currently taking? | TDF+3TC/FTC+LPV/r 1  TDF+3TC/FTC+EFV 2  AZT+3TC+LPV/r 3  AZT+3TC+EFV 4  Other: 5 |
| EH8 | Do you take other pills other than ARVs? | No 0  Yes 1 |
| EH9 | How many other pills do you take per day? |  |

| **ANTIRETROVIRAL THERAPY KNOWLEDGE** | | | | |
| --- | --- | --- | --- | --- |
|  |  | **Yes** | **No** | **Don’t know** |
| KN1 | HIV can be prevented by ART after rape | 1 | 0 | 98 |
| KN2 | HIV can be controlled by ART | 1 | 0 | 98 |
| KN3 | HIV can be cured by ART | 1 | 0 | 98 |
| KN4 | Taking ART prevents disease progression | 1 | 0 | 98 |
| KN5 | Not starting ART when indicated can make you sick | 1 | 0 | 98 |
| KN6 | Antiretroviral medicine can cause side effects | 1 | 0 | 98 |
| KN7 | Taking ART on schedule prevents you from being sick | 1 | 0 | 98 |
| KN8 | Missing doses of ART leads to disease progression | 1 | 0 | 98 |
| KN9 | Missing doses of ART increases the risk of transmitting HIV to a partner | 1 | 0 | 98 |
| KN10 | Missing doses of ART during pregnancy increases the risk of transmitting HIV to the unborn baby | 1 | 0 | 98 |
| KN11 | ART prevent mother-to-child HIV transmission | 1 | 0 | 98 |
| KN12 | When ART is started in pregnancy is important in reducing the risk of mother-to-child transmission. | 1 | 0 | 98 |

| **REASONS FOR MISSING DOSES** | | | | | |
| --- | --- | --- | --- | --- | --- |
|  |  | **Never** | **Rarely** | **Sometimes** | **Often** |
| RD1 | In the past month, how often have you missed taking your medications? | **0** | **1** | **2** | **3** |
| *You missed taking your medications because you:* | | | | | |
| RD1 | Were away from home? | 0 | 1 | 2 | 3 |
| RD2 | Were busy with other things? | 0 | 1 | 2 | 3 |
| RD3 | Simply forgot? | 0 | 1 | 2 | 3 |
| RD4 | Had too many pills to take? | 0 | 1 | 2 | 3 |
| RD5 | Wanted to avoid side effects? | 0 | 1 | 2 | 3 |
| RD6 | Did not want others to notice you taking medication? | 0 | 1 | 2 | 3 |
| RD7 | Had a change in daily routine? | 0 | 1 | 2 | 3 |
| RD8 | Felt like the drug was toxic/harmful? | 0 | 1 | 2 | 3 |
| RD9 | Fell asleep/slept through dose time? | 0 | 1 | 2 | 3 |
| RD10 | Felt sick or ill? | 0 | 1 | 2 | 3 |
| RD11 | Felt depressed/overwhelmed? | 0 | 1 | 2 | 3 |
| RD12 | Had problem taking pills at specific times? | 0 | 1 | 2 | 3 |
| RD13 | Ran out of pills? | 0 | 1 | 2 | 3 |
| RD14 | Felt good? | 0 | 1 | 2 | 3 |

| **DISCLOSURE OF HIV STATUS (Mother)**  *Interviewer reads: Please tell me if you have shared your HIV positive status with the following category of people* | | | | | | | |
| --- | --- | --- | --- | --- | --- | --- | --- |
|  |  | **No** | | **Yes** | | **N/A** | |
| DIS1 | Your current partner | 0 | | 1 | | 98 | |
| DIS2 | Close relatives | 0 | | 1 | | 98 | |
| DIS3 | Your children | 0 | | 1 | | 98 | |
| DIS4 | Your colleagues | 0 | | 1 | | 98 | |
| DIS5 | Your friends | 0 | | 1 | | 98 | |
| **REASONS FOR DISCLOSING**  *Interviewer reads: How are important are the following reasons to tell people that you are HIV+…* | | | | | | | |
|  |  | **Not at all important** | **Somewhat important** | | **Very important** | | **N/A** |
| RFD1 | It is too hard to keep it a secret? | 0 | 1 | | 2 | | 98 |
| RFD2 | To receive more social support? | 0 | 1 | | 2 | | 98 |
| RFD3 | To be able to talk about your feelings and problems? | 0 | 1 | | 2 | | 98 |
| RFD4 | To receive more help? | 0 | 1 | | 2 | | 98 |
| RFD5 | To help others avoid getting HIV? | 0 | 1 | | 2 | | 98 |
|  |  |  |  | |  | |  |
| **DISCLOSURE OF HIV STATUS (infant)**  *Interviewer reads: Please tell me if you have shared your baby’s HIV status with the following category of people* | | | | | | | |
| DIS6 | Your current partner | 0 | | 1 | | 98 | |
| DIS7 | Close relatives | 0 | | 1 | | 98 | |
| DIS8 | Other caregivers | 0 | | 1 | | 98 | |

| **DEPRESSION** *Interviewer read: Now I will read a list of some of the ways you may have felt or behaved within the past week. Please tell me how often during the past week have you …* | | | | | |
| --- | --- | --- | --- | --- | --- |
|  |  | **No days** | **1-2 days** | **3-4 days** | **5-7 days** |
| DEP1 | Felt that you could not shake off the blues even with help from your family or friends | 0 | 1 | 2 | 3 |
| DEP2 | Felt depressed | 0 | 1 | 2 | 3 |
| DEP3 | Thought your life has been a failure | 0 | 1 | 2 | 3 |
| DEP4 | Been restless while sleeping | 0 | 1 | 2 | 3 |
| DEP5 | Had crying spells. | 0 | 1 | 2 | 3 |
| DEP6 | Felt bothered by things that usually don’t bother you | 0 | 1 | 2 | 3 |
| DEP7 | Did not feel like eating, you appetite was poor | 0 | 1 | 2 | 3 |
| DEP8 | Had trouble keeping your mind on what you were doing | 0 | 1 | 2 | 3 |
| DEP9 | Felt that people disliked you | 0 | 1 | 2 | 3 |

| **PERCEIVED SOCIAL SUPPORT***Interviewer reads: Please indicate* ***how many*** *people you feel provide each type of support and* ***how generally satisfied*** *you are with the support. Circle one number on each line.* | | | | | | | |
| --- | --- | --- | --- | --- | --- | --- | --- |
|  | **Whom can you really count on** | **#** | **Very****satisfied** | **Satisfied** | **Neutral** | **Dissatisfied** | **Very**  **dissatisfied** |
| PSS1 | To be dependable when you need help? | _ _ _ | 5 | 4 | 3 | 2 | 1 |
| PSS2 | To help you feel more relaxed when you are under pressure? | _ _ _ | 5 | 4 | 3 | 2 | 1 |
| PSS3 | To accept you totally, including both your worst and your best points? | _ _ _ | 5 | 4 | 3 | 2 | 1 |
| PSS4 | To care about you, regardless of what is happening to you? | _ _ _ | 5 | 4 | 3 | 2 | 1 |
| PSS5 | To help you feel better when you are feeling generally down? | _ _ _ | 5 | 4 | 3 | 2 | 1 |
| PSS6 | To console you when you are very upset? | _ _ _ | 5 | 4 | 3 | 2 | 1 |

| **TB**  *Interviewer reads: Now I would like to ask you some questions about tuberculosis.* | | | | |
| --- | --- | --- | --- | --- |
|  |  | **Yes** | **No** | **Don’t know** |
| TB1 | Have you ever been diagnosed with TB? | 1 | 0 | 98 |
| TB2 | Did you get treatment for TB when you had TB? | 1 | 0 | 98 |
| TB3 | Did you complete your treatment for TB? | 1 | 0 | 98 |
| TB4 | Are you currently receiving treatment for TB? | 1 | 0 | 98 |
